# From Pigment Chemistry to Nanomaterials: Fungal Pigments as Reducing and Stabilizing Agents in Green Nanoparticle Synthesis

**DOI:** 10.3390/microorganisms14040792

**Published:** 2026-03-31

**Authors:** Akshay Chavan, Guruprasad Mavlankar, Umesh B. Kakde, Laurent Dufossé, Sunil Kumar Deshmukh

**Affiliations:** 1 The Institute of Science, Dr. Homi Bhabha State University, 15, Madame Cama Road, Mumbai 400032, India; akshaychavan1719@gmail.com (A.C.); guru1996mav@gmail.com (G.M.); 2 Chimie et Biotechnologie des Produits Naturels (CHEMBIOPRO Lab) & ESIROI Agroalimentaire, Université de la Réunion, 15 Avenue René Cassin, 97744 Saint-Denis, France; 3 TERI-Deakin Nano Biotechnology Centre, The Energy and Resources Institute, Darbari Seth Block, IHC Complex, Lodhi Road, New Delhi 110003, India; sunildeshmukh1958@gmail.com

**Keywords:** fungal pigment, natural pigments, nanoparticles, green synthesis, secondary metabolites

## Abstract

Fungal pigments have gained attention as eco-friendly and versatile materials for green nanotechnology because of their varied chemical structures, inherent redox properties, and strong metal ion-binding capabilities. These pigments, such as polyketides, azaphilones, melanins, and carotenoids, can function simultaneously as reducing, capping, and surface-functionalizing agents, facilitating the environmentally friendly production of metallic nanoparticles without the use of harmful chemicals. This review provides a critical overview of recent progress in the production, extraction, and application of fungal pigments for nanoparticle synthesis, focusing on the mechanistic roles of pigment functional groups in metal ion reduction, nanoparticle nucleation, growth, and stabilization. The impact of pigment chemistry and reaction conditions on the nanoparticle size, shape, crystallinity, and colloidal stability was thoroughly examined. Additionally, this review highlights the emerging biomedical, environmental, and industrial applications of pigment-mediated nanoparticles, emphasizing their biocompatibility and functional adaptability. Key challenges, such as variability in pigment yield and composition, limited mechanistic validation, lack of standardized synthesis protocols, and insufficient toxicity assessment, are critically analyzed in this review. Finally, future directions are outlined, emphasizing the importance of process optimization, omics-guided pigment discovery, and comprehensive safety evaluations as crucial steps toward the scalable and reliable use of fungal pigment-mediated nanoparticle synthesis in sustainable nanotechnology.

## 1. Introduction

Natural pigments derived from microorganisms have attracted significant interest from both scientific and industrial communities because of their numerous advantages, including structural diversity, compatibility with biological systems, and environmentally friendly production methods [1,2,3]. These pigments offer a promising alternative to synthetic dyes, which are often associated with environmental and health hazards owing to their toxic residues, persistence in the environment, and reliance on petrochemical sources [4,5]. In contrast, pigments obtained from bacteria [6], fungi [7], yeasts [8], cyanobacteria, and microalgae [9] exhibit a wide range of chemical structures, enabling a variety of colors and applications in industries such as food, cosmetics, textiles, pharmaceuticals, and packaging [10,11,12,13].

Fungal pigments represent a rich and largely untapped reservoir of bioactive compounds produced through complex secondary metabolic processes [14]. Fungi, especially those belonging to the genera *Penicillium*, *Aspergillus*, *Monascus*, *Trichoderma*, and *Fusarium*, generate a wide variety of polyketides, azaphilones, anthraquinones, melanins, and carotenoids, each with unique chromophoric characteristics and functional groups [15,16,17]. These structural features not only contribute to coloration but also offer redox activity, metal-binding capabilities, and chemical stability, positioning fungal pigments as promising candidates for developing eco-friendly and sustainable materials [5,15].

Simultaneously, nanotechnology has rapidly progressed towards environmentally friendly synthesis techniques that eliminate the need for hazardous reducing agents, harsh reaction conditions, and non-renewable chemical inputs [18]. Biogenic or “green” synthesis methods have emerged as viable alternatives, facilitating the production of metal and metal-oxide nanoparticles using biological molecules as reducing and stabilizing agents [19,20,21,22].

Among the various biological resources studied, the synthesis of nanoparticles using fungal pigments has garnered significant attention [23,24,25]. These pigments possess natural electron-donating abilities, phenolic and quinone groups, and surface-active characteristics, which allow them to reduce metal ions and stabilize the resulting nanoparticles simultaneously [14,26]. This dual functionality provides a distinct advantage over other eco-friendly methods, which typically require separate agents for reduction and capping processes. Recognizing that fungal pigments have different levels of reactivity is crucial, as their effectiveness in synthesizing nanoparticles largely depends on their unique chemical structures and functional groups [23].

The growing interest in employing fungal pigments for nanoparticle synthesis is motivated by several reasons: (i) the ability to generate substantial quantities of pigments through fermentation, which supports scalability [16,27]; (ii) the diverse structures of these pigments, which affect the shape and reactivity of the nanoparticles [28]; (iii) a reduced environmental impact compared to traditional chemical methods [29,30]; and (iv) the potential to produce biocompatible, functionalized nanoparticles suitable for various applications [31,32].

Despite notable advancements in this area, the field still faces considerable challenges. Issues such as variability in pigment production, the complexity of purification techniques [33], limited understanding of pigment-metal interactions [34], and lack of standardized synthesis parameters impede reproducibility [35]. Furthermore, comprehensive evaluations of nanoparticle stability, cytotoxicity, and environmental impact are insufficient, limiting their potential for broad application [36].

As this interdisciplinary domain grows swiftly, it is crucial to undertake a thorough and critical assessment of nanoparticle synthesis enabled by fungal pigments. This review offers a detailed examination of the wide variety of fungal pigments, the techniques for their extraction and characterization, insights into the processes involved in nanoparticle biosynthesis, the physicochemical attributes of the resulting nanomaterials, and their innovative applications. Additionally, it discusses existing challenges, technological gaps, and future research directions to aid in the development of robust, scalable, and sustainable pigment-based nanotechnologies.

### Literature Search Strategy

The literature review for this study was conducted utilizing prominent scientific databases, including Scopus, Web of Science, PubMed, and Google Scholar. The selection of articles was guided by keywords such as “fungal pigments,” “green synthesis of nanoparticles,” “biosynthesis of nanoparticles,” and “pigment-mediated nanoparticle synthesis.” Only peer-reviewed articles written in English were considered. The review concentrated on research examining the production, extraction, characterization, and application of fungal pigments in nanoparticle synthesis. Recent publications were prioritized to emphasize the latest advancements in the field. Additional references were also obtained from the bibliographies of the selected articles.

## 2. Fungal Pigments: Origins, Composition, and Biological Activity

Fungi rank among the most chemically varied organisms that produce pigments, creating a wide range of secondary metabolites with distinct colors and functional characteristics [28,37,38]. In contrast to plant pigments, which are often limited by seasonal factors and agricultural constraints, fungal pigments can be generated throughout the year under controlled fermentation settings [27,39]. This ability allows for consistent production, scalability, and the ability to adjust the composition, offering significant benefits for sustainable biotechnology and nanotechnology applications [40,41,42].

The pigments under consideration encompass a diverse array of chemical classes, including carotenoids, melanins, azaphilones, quinones, flavins, anthraquinones, and naphthoquinones [43,44]. These compounds are responsible for a spectrum of colors and fulfill various biological functions, such as antimicrobial, antioxidant, anticancer, and immunomodulatory activities [45]. This multifunctional nature elevates fungal pigments from mere colorants to bioactive agents of significant interest for diverse industrial applications [15,41,46].

### 2.1. Fungal Pigments Sources

Filamentous fungi, yeasts, and higher fungi are widely recognized for pigment production, as summarized in Table 1 [17,47,48]. These fungi generate pigments either inside or outside their cells as part of their secondary metabolism, which is often associated with their function in shielding against oxidative stress and light damage, acting as secondary protective metabolites [16]. The yield and intensity of pigment production are significantly influenced by nutritional and environmental factors, including carbon and nitrogen sources, pH, temperature, light, moisture, and oxygen levels [49]. For instance, pigment production in filamentous fungi is increased when grown in complex media such as potato dextrose and malt extract [50].

Fungal pigments generated by fungi from terrestrial, marine, and endophytic environments display unique characteristics shaped by their ecological settings and metabolic processes. Terrestrial fungi, including those from the *Aspergillus*, *Fusarium*, *Penicillium*, and *Trichoderma* genera, mainly produce aromatic polyketide pigments, such as melanins, quinones, flavins, ankaflavin, anthraquinone, and naphthoquinone [56].

Fungi sourced from marine environments, such as certain *Talaromyces* species, often generate highly oxygenated polyketide pigments with intricate structures, including monascus-like azaphilone compounds. These compounds can undergo halogenation and exhibit enhanced redox characteristics. These pigments typically exhibit bright colors ranging from red to yellow and have bioactive properties that are useful in the dyeing, cosmeceutical, and food industries [17,97,98].

Endophytic fungi isolated from medicinal plants often produce pigments that closely resemble the metabolites of their host plants, frequently displaying notable bioactivities, such as antimicrobial, antioxidant, and anticancer effects [99]. For instance, the endophytic strain *Talaromyces assiutensis* generates extracellular red pigments with strong antibacterial properties against pathogens such as *Staphylococcus aureus* and methicillin-resistant *S. aureus*, as well as anticancer activity against HeLa cells [99]. These pigments may share biosynthetic pathways or chemical structures with their plant hosts, underscoring the metabolic interactions and adaptations within the endophytic environment [100,101,102].

Recent advancements in fungal pigment production have been propelled by screening various strains to identify those that produce high quantities of unique pigments in diverse environments, both terrestrial and marine [16,50]. Methods such as mutagenesis and genetic engineering have been employed to boost pigment yields and ensure safety by reducing the simultaneous production of mycotoxins [103]. Furthermore, omics-guided approaches, such as genomics, transcriptomics, and metabolomics, contribute to the understanding of biosynthetic pathways, enabling metabolic engineering to optimize specific pigment synthesis and scale up production in controlled fermentation environments [16,50].

### 2.2. Major Classes of Fungal Pigments

Fungal pigments represent a diverse group of chemically distinct secondary metabolites synthesized through biosynthetic pathways originating from polyketides, terpenoids (isoprenoids), and shikimates or amino acid-derived pathways [5]. Each of these pathways imparts unique structural, optical, and functional characteristics. The molecular configurations of these compounds, which include conjugated polyenes, phenolic hydroxyls, carbonyls, quinonoid systems, and heterocyclic elements, influence their coloration, redox properties, and metal-binding capabilities [5].

Fungal pigments are broadly categorized into three major biosynthetic groups: (i) terpenoid pigments, such as carotenoids; (ii) pigments derived from polyketides, including anthraquinones, azaphilones, and naphthoquinones; and (iii) nitrogen-containing metabolites originating from amino acid or nucleotide precursors, such as riboflavin [28,104,105,106,107]. It is important to recognize that certain pigment groups, such as quinones and anthraquinones, are not independent categories but rather belong to the broader class of polyketide-derived metabolites [108,109]. Furthermore, melanins constitute a biosynthetically diverse group, with DHN-melanins being synthesized via polyketide pathways and DOPA-melanins resulting from tyrosine metabolism [110]. The subsequent sections will provide a detailed examination of the primary classes of fungal pigments based on their biosynthetic origins. This classification functions as a general biosynthetic framework rather than a comprehensive categorization. The subsequent sections provide in-depth analyses of specific pigment subclasses, including melanins, anthraquinones, azaphilones, and quinones, emphasizing their structural and functional significance.

Understanding the chemical diversity of fungal pigments is essential, as they exhibit varying chemical reactivity and the capacity to interact with metals. The functional properties of these pigments are primarily determined by their molecular structure and the presence of redox-active groups [111]. For example, polyketide-derived pigments, such as anthraquinones and azaphilones, as well as melanins, which contain phenolic, hydroxyl, and quinonoid components, generally demonstrate enhanced redox activity and stronger interactions with metal ions [112]. In contrast, other pigment classes, including certain carotenoids and riboflavin, may display limited reactivity due to their redox potential, polarity, and solubility [113]. These variations in chemical structure and redox behavior directly influence the suitability of different pigment classes for nanoparticle synthesis, as discussed in Section 4.

#### 2.2.1. Fungal Carotenoids

Carotenoids represent a significant group of fungal pigments within the terpenoid category, distinguished by a C_40_ isoprenoid structure and a series of extended conjugated double bonds [114]. These conjugated double bonds are mainly responsible for their optical characteristics, which range in color from yellow to deep red, as well as their strong antioxidant properties and photoprotective roles, particularly in neutralizing reactive oxygen species and reducing photo-oxidative harm [115,116]. In fungi, carotenoid production is frequently triggered by environmental conditions, such as light exposure, especially blue light, which promotes carotenogenesis by activating pathway-specific genes at the transcriptional level [114].

Fungal carotenoids are categorized into two main groups based on their chemical composition: oxygen-free carotenes, such as β-carotene, lycopene, and torulene, and oxygenated xanthophylls, including astaxanthin, canthaxanthin, and torularhodin [117]. The level and presence of oxygenation play crucial roles in determining redox properties, polarity, and interactions with metal ions and biological membranes [15]. Table 2 presents a list of carotenoids, along with their structures and biological properties.

#### 2.2.2. Fungal Polyketides

Fungal polyketide pigments represent one of the most structurally varied groups of fungal pigments, primarily synthesized by iterative type I polyketide synthases (PKSs) [146]. These large multifunctional megasynthases (i.e., large, multi-domain enzymatic complexes that catalyze multiple sequential reactions within a single protein assembly) construct carbon skeletons through repeated catalytic cycles [147,148]. PKSs produce a wide range of polyketide backbones, which are further modified by tailoring enzymes, such as oxygenases, reductases, and cyclases, to form complex structures [105,149]. The iterative catalytic process enables PKSs to manage the regiospecific cyclization and folding of poly-beta-ketone intermediates, determining the final structure and functional characteristics of pigments [150]. This group of pigments encompasses several structurally distinct subclasses, including melanins, anthraquinones, azaphilones, quinones, and naphthoquinones [14]. While the majority of these pigments are derived from polyketides, melanins demonstrate biosynthetic diversity. For example, DHN-melanins are synthesized from polyketides, whereas DOPA-melanins are produced from tyrosine [151]. Many of these pigments contain redox-active functional groups and, in certain cases, polymeric structures that are integral to advancements in green nanotechnology [105]. Table 3 presents a list of polyketides, along with their structures and biological properties.

#### 2.2.3. Fungal Riboflavin and Related Nitrogen-Containing Pigments

Fungi and many other microbes generate riboflavin, a yellow, water-soluble vitamin. Rather than using classic chemical synthesis methods, advanced biotechnological methods are being used to synthesize riboflavin [167]. Microorganisms, such as the ascomycete *Ashbya gossypii* and the yeast *Candida famata*, predominantly synthesize riboflavin through biotechnological methods that are commercially viable [168,169]. The most frequently used strain for yield and genetic stability is *A. gossypii*. Riboflavin is distinguished by a conjugated heterocyclic ring system that participates in redox reactions [170,171]. Nevertheless, its redox characteristics are more regulated and less adaptable compared to those of phenolic or quinonoid polyketide pigments.

Consequently, riboflavin and analogous nitrogen-containing pigments generally exhibit limited efficacy in directly reducing metal ions during nanoparticle synthesis. When involved, their role is more frequently associated with electron mediation, coordination interactions, or stabilization, rather than functioning as primary reducing agents [172]. Therefore, in comparison to pigments derived from polyketides and melanins, their contribution to nanoparticle formation is relatively minor and contingent upon the specific system.

Not all fungal pigments exhibit equal efficacy in nanoparticle synthesis. Pigments that lack redox-active functional groups or are predominantly hydrophobic, such as certain carotenoids, may demonstrate limited effectiveness in reducing and stabilizing metal ions due to insufficient interaction with aqueous metal precursors [173]. Therefore, the selection of pigment should be informed by its chemical functionality, solubility, and capacity to bind with metals.

### 2.3. Extraction and Recovery of Fungal Pigments

Fungal pigments can be extracted using either traditional solvent-based techniques or modern eco-friendly methods, depending on factors such as pigment polarity, cellular location, and intended use [174,175]. Common solvents used in these processes include ethanol, methanol, ethyl acetate, and water, often in combination with cell disruption methods such as ultrasonication or enzymatic treatment to facilitate the release of pigments from within cells [176,177]. Recently, environmentally friendly techniques such as ultrasound-assisted extraction, pressurized liquid extraction, and aqueous two-phase systems have gained popularity owing to their lower solvent requirements, increased extraction efficiency, and improved pigment purity [178,179].

**Figure 1:**
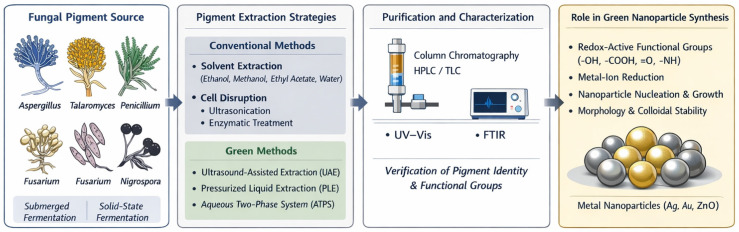
Schematic illustration of fungal pigment extraction and recovery strategies for nanoparticle synthesis. Pigments produced by diverse fungal genera are recovered using conventional solvent-based or eco-friendly extraction techniques, followed by purification and spectroscopic characterization. Preservation of redox-active functional groups is essential for metal-ion reduction, nanoparticle nucleation, growth, morphology control, and colloidal stability in green synthesis systems. Arrows indicate the sequential workflow of the process, while different color panels represent distinct stages, including pigment source, extraction, purification, and nanoparticle synthesis.

The extraction method plays a crucial role in determining the pigment composition, redox properties, and availability of functional groups, which are vital for applications such as nanoparticle synthesis [180]. Organic solvents are generally favored for extracting intracellular polyketide and quinone pigments, whereas aqueous systems are effective for recovering extracellular or hydrophilic pigments from fermentation broths [14,181]. Chromatographic techniques are typically used for further purification, and spectroscopic methods, such as UV–Vis and FTIR, are utilized to verify pigment identity and the integrity of functional groups (Figure 1) [169,181,182]. It is crucial to maintain redox-active and coordinating moieties during extraction, as these chemical characteristics are directly responsible for metal-ion reduction, nanoparticle nucleation, growth, morphology, and colloidal stability in green synthesis systems [183].

## 3. Green Synthesis of NPs

Green synthesis employs biological systems to create and stabilize nanoparticles (NPs), offering environmentally friendly and sustainable alternatives for nanoparticle production [184]. The formation of nanoparticles is facilitated by bacteria, yeasts, filamentous fungi, plant extracts, and agricultural waste biomass through enzymatic redox processes and biomolecule-mediated capping [185,186,187]. Bacteria and fungi utilize reductases, peptides, polysaccharides, and bioactive pigments to aid in the reduction in metal ions [5,23,188,189], whereas plant-derived phytochemicals such as phenolics, flavonoids, terpenoids, and alkaloids enable rapid NP formation at ambient temperature [190,191,192,193,194]. Additionally, biomass residues such as fruit peels, tea waste, and sugarcane bagasse can facilitate cost-effective green synthesis methods [195,196,197].

Fungi are metabolically versatile eukaryotes capable of producing a wide array of extracellular enzymes and secondary metabolites in response to environmental stress, including exposure to toxic metal ions [198,199,200,201]. Among these metabolites, fungal pigments play a central role because of their strong redox activity, metal-chelating capacity, and antioxidant properties [24]. These characteristics enable fungi to detoxify metal ions by converting them into stable metallic nanoparticles through intracellular or, more efficiently, extracellular pathways. In extracellular systems, fungal pigments act in concert with secreted enzymes, proteins, and polysaccharides to drive metal-ion reduction while simultaneously capping and stabilizing the resulting nanoparticles [202]. The diversity of fungal pigment chemistry and secretion profiles across species allows for controlled and eco-friendly nanoparticle synthesis, positioning fungi as sustainable biofactories for green nanotechnology.

## 4. Mechanism of Nanoparticle Synthesis Facilitated by Fungal Pigments

To elucidate the roles of fungal pigments in nanoparticle synthesis, it is essential to differentiate between the reduction and stabilization processes. The conversion of metal ions into zero-valent nanoparticles is facilitated by electron transfer from redox-active functional groups, such as phenolic hydroxyl and quinonoid moieties. This conversion primarily involves the oxidation of these groups while concurrently reducing the metal ions [203]. Phenolic hydroxyl groups, characterized by their electron-rich O-H bond, can donate electrons through mechanisms such as single-electron transfer followed by proton transfer (SET-PT), hydrogen atom transfer (HAT), or sequential proton-loss electron transfer (SPLET). This electron donation results in the formation of phenoxy radicals or quinonoid structures upon oxidation. Consequently, the electron donation reduces metal ions (e.g., Ag^+^, Au^3+^, Fe^3+^) to their elemental zero-valent metallic forms (Ag^0^, Au^0^, Fe^0^), thereby initiating the nucleation of metal nanoparticles [204,205].

Following reduction, it is imperative to stabilize nanoparticles to prevent agglomeration and preserve their colloidal state. Fungal pigments and associated biomolecules adhere to the surfaces of nanoparticles, forming a capping layer that imparts electrostatic repulsion and steric hindrance [206,207,208]. These processes are governed by fundamental physicochemical principles, including redox reactions, surface charge interactions, and interfacial adsorption mechanisms.

Fungal pigments, including polyketides, anthraquinones, melanins, and carotenoids, exhibit unique redox properties influenced by their structural makeup. Phenolic hydroxyl groups can donate electrons or hydrogen atoms, whereas carbonyl and quinonoid groups serve as electron acceptors, enabling these pigments to function effectively as redox shuttles [15,109,209,210]. Melanins, characterized by their polymeric and multifunctional structures, exhibit strong metal-binding and surface passivation properties, whereas carotenoids excel as radical scavengers owing to their extensive conjugated polyene systems [27]. These features collectively facilitate the controlled reduction in metal ions and influence nanoparticle growth dynamics.

Recent advances in fungal nanobiotechnology have highlighted the potential of incorporating fungal pigments in nanomaterial synthesis as a viable approach for creating sustainable and biocompatible nanoparticles [14]. Pigment-mediated nanostructures have shown enhanced stability, increased bioavailability, and adjustable surface characteristics, which support their use in drug delivery, biosensing, food preservation, and cosmetic products [211]. In contrast to their chemically synthesized counterparts, pigment-assisted nanomaterials reduce environmental impact by avoiding toxic reagents and utilizing renewable biological resources [212].

Fungi serve as effective biofactories by releasing pigments, proteins, and other organic metabolites into their surrounding environment, which collectively impact the formation of nanoparticles. Pigments facilitate the reduction in metal ions, while the biomolecules secreted alongside them act as capping and stabilizing agents, preventing the aggregation of nanoparticles and allowing control over their size, shape, and colloidal stability under mild reaction conditions [213,214]. The effectiveness of this process is influenced by the inherent chemical characteristics of the pigments and the physiological conditions of the fungal system, which dictate the composition and concentration of the secreted biomolecules [25,215].

The synthesis of nanoparticles mediated by fungal pigments is a chemically driven but biologically controlled process, in which the structure of the pigment, its redox potential, and its surface affinity play crucial roles in the nucleation, growth, and stabilization of nanoparticles. This mechanistic approach lays the groundwork for comprehending how reaction parameters and pigment chemistry can be strategically adjusted to achieve controlled and environmentally friendly nanoparticle synthesis, as discussed in the following sections.

### 4.1. Role of Pigments as Reducing Agents

Fungal pigments facilitate nanoparticle synthesis predominantly through electron transfer processes driven by redox reactions, wherein chemically active groups convert metal ions into their zero-valent nanoscale form [216]. Phenolic hydroxyl (–OH) groups play a pivotal role in reducing metal ions via electron transfer or hydrogen atom transfer mechanisms. These groups can undergo reversible oxidation, thereby enabling the transformation of metal ions (e.g., Ag^+^, Au^3+^) into their zero-valent states [217]. In contrast, functional groups such as carbonyl (C=O), methoxy, and amino groups do not act as strong reducing agents independently but may indirectly contribute by modifying electronic properties or participating in coordination interactions with metal ions [218,219]. Consequently, only specific pigment classes containing phenolic or similar electron-donating groups serve as effective reducing agents, while other pigments may be redox-inactive or primarily contribute to stabilization rather than reduction.

**Figure 2:**
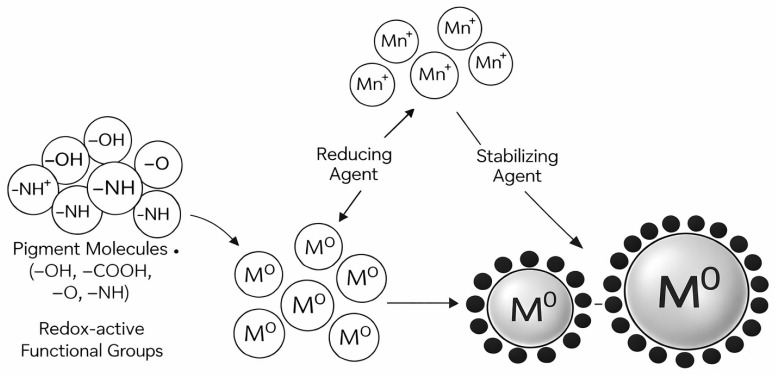
Mechanism of pigment-mediated synthesis of metal nanoparticles via reduction, nucleation, and stabilization driven by redox-active functional groups. Arrows indicate the direction and sequence of the process involved in nanoparticle synthesis.

Metal-ion reduction occurs via a combination of synergistic single-electron transfer (SET) and hydrogen atom transfer (HAT) pathways. In SET processes, electrons are transferred directly from pigment molecules to metal precursors, such as Ag^+^, Au^3+^, and Cu^2+^. HAT involves the donation of hydrogen from phenolic or similar groups, resulting in the formation of reduced metal atoms and oxidized pigment intermediates (Figure 2). These pathways often operate simultaneously, particularly in pigments rich in phenolic structures and other electron-donating groups, which enhance reduction efficiency and expedite reaction kinetics [220,221].

**Figure 3:**
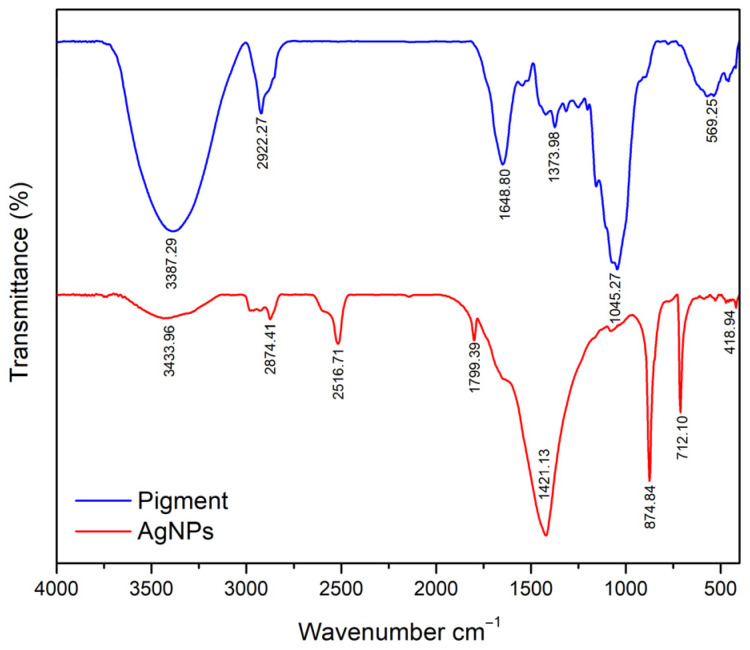
Representative FTIR spectra illustrating functional group involvement in fungal pigment–mediated silver nanoparticle synthesis, reproduced from Chavan et al. (2026) [24] under a CC BY 4.0 license.

Spectroscopic data provide compelling evidence for the role of pigments in metal reduction processes. FTIR analyses often reveal changes in the position and intensity of bands related to –OH, C=O, and –NH groups after nanoparticle formation, indicating their participation in electron donation and metal-ion transformation processes [222,223]. These spectral trends are in close agreement with our previously reported FTIR analysis of pigment-derived AgNPs, which showed systematic band shifts and intensity changes indicative of redox-driven metal reduction and surface coordination [24]. Figure 3 provides a representative FTIR example demonstrating functional group participation during pigment-mediated nanoparticle formation.

Importantly, the release of fungal pigments into the extracellular environment facilitates the synthesis of nanoparticles without disrupting the biomass. This method offers clear benefits over bacterial and plant-based systems, particularly in terms of scalability, process simplicity, and sustainability [224].

### 4.2. Role of Pigments as Capping and Stabilizing Agents

In addition to their basic role, fungal pigments serve as effective capping and stabilizing agents that influence the size, shape, and colloidal stability of nanoparticles. These pigment molecules contain various functional groups, such as aromatic rings, phenolic hydroxyls, carboxylates, polysaccharide-linked moieties, and protein-like residues, which easily adhere to nanoparticle surfaces through electrostatic interactions, hydrogen bonds, and π–metal coordination. This pigment layer, bound to the surface, creates a biological corona that provides steric and electrostatic repulsion, successfully preventing nanoparticle aggregation, even in environments with high ionic strength [225,226].

Various types of fungal pigments have distinct stabilization characteristics. Pigments rich in quinones, typically produced by representatives of the genera such as *Aspergillus*, *Penicillium*, and *Fusarium*, possess extended conjugated systems that facilitate surface adsorption, often resulting in spherical nanoparticles with narrow size distributions [15,108]. In contrast, melanin, a highly polymeric and diverse pigment, forms thick, multifunctional layers around nanoparticles, providing remarkable colloidal stability but frequently leading to wider size distributions owing to its multiple binding modes and structural diversity [227,228,229,230,231].

Azaphilones, a structurally diverse group of oxygenated fungal polyketides, exhibit significant capping and shape-directing properties owing to their pyranoquinone core and numerous coordination sites. These pigments not only stabilize the nanoparticles but also influence anisotropic growth pathways, resulting in non-spherical shapes such as triangular, hexagonal, or other faceted forms, especially in nanoparticles created with pigments derived from Monascus [14,232,233,234].

Fungal pigments collectively act as natural nano-architects, stabilizing newly formed nanoparticles and determining their ultimate size and shape without the need for synthetic surfactants or external capping agents.

The functional significance of fungal pigments is predominantly determined by their chemical classification. Pigments derived from polyketides, particularly anthraquinones, function as both reducing and stabilizing agents due to the presence of quinone and phenolic groups, which facilitate electron transfer and surface coordination. In contrast, melanins primarily serve as stabilizing agents owing to their polymeric structure and numerous binding sites, which enhance strong surface adsorption and effectively prevent nanoparticle aggregation. Although carotenoids exhibit antioxidant properties, their direct involvement in metal ion reduction is generally limited, contributing mainly through radical scavenging mechanisms. Azaphilone pigments primarily act as stabilizing and shape-directing agents due to their oxygenated heterocyclic structures. Conversely, riboflavin primarily functions as a reducing agent, attributed to its redox-active isoalloxazine ring system that facilitates efficient electron transfer.

### 4.3. Optimization Strategies for Pigment-Mediated Nanoparticle Synthesis

Optimizing the synthesis of nanoparticles through pigments is crucial for controlling factors such as nanoparticle yield, size distribution, shape, surface properties, and colloidal stability, all of which influence their effectiveness in biomedical, catalytic, sensing, and environmental applications [14,235,236]. Since fungal pigments serve as both reducing and capping agents, optimization approaches need to incorporate both the biological production of pigments and the physicochemical processes involved in nanoparticle formation [14,178,237].

At the biological level, the parameters for cultivating fungi, especially the sources of carbon and nitrogen, have a direct impact on pigment composition, redox potential, and the availability of functional groups, which in turn affect the efficiency of metal-ion reduction and the density of nucleation [14,16,216,236]. Carbon sources like glucose, sucrose, glycerol, and agro-industrial residues influence the pathways of pigment biosynthesis and the capacity to donate electrons, while the availability of nitrogen controls the enzymatic activity related to pigments and the profiles of secondary metabolites, which together dictate the kinetics of nanoparticle formation [238,239]. These factors not only determine the yield of nanoparticles but also affect the uniformity and stability of the nanostructures produced [240].

Critical parameters such as pH, temperature, incubation duration, pigment concentration, and the amount of metal precursor are essential in regulating the nucleation and growth of nanoparticles. These factors significantly influence the size, morphology, and stability of nanoparticles. In optimization strategies, these variables are systematically adjusted and refined to achieve the desired nanoparticle characteristics, rather than being reinterpreted in a mechanistic manner [241,242,243].

Reaction time plays a crucial role in the development of nanoparticles, as extended incubation can lead to particle merging and Ostwald ripening, resulting in wider size distributions and decreased colloidal stability. Therefore, it is essential to precisely control the reaction time to halt growth at the desired nanoparticle size and shape [244,245]. The synthesis of nanoparticles is profoundly affected by the concentration of pigment and the pigment-to-metal ratio, which are critical variables for optimization. Rather than reinterpreting their mechanistic roles, these factors are experimentally adjusted to ensure a balance in nucleation, controlled growth, and the desired stability of the colloid [246]. To systematically optimize these interrelated factors, statistical methods like one-factor-at-a-time (OFAT) screening and response surface methodology (RSM) are increasingly utilized [49,247,248].

OFAT approaches are beneficial for initial parameter identification, while RSM facilitates multivariate optimization by accounting for interaction effects among biological and reaction parameters [249,250]. RSM designs, such as central composite and Box–Behnken models, produce predictive equations that link synthesis conditions to nanoparticle characteristics like size, dispersity, crystallinity, and surface charge. Recent developments have further combined RSM with machine learning tools, including artificial neural networks, to improve predictive accuracy and enhance process robustness in the synthesis of biogenic nanoparticles [251,252,253].

From a scale-up perspective, optimized pigment-mediated nanoparticle synthesis benefits substantially from controlled bioreactor-based cultivation systems, which allow precise regulation of pH, temperature, oxygen availability, and nutrient supply. Strategies such as fed-batch fermentation and standardized downstream processing ensure consistent pigment secretion and reproducible nanoparticle characteristics at larger volumes [254]. Collectively, optimization strategies form a critical bridge between fungal pigment chemistry and the rational, scalable, and sustainable production of nanoparticles with tunable physicochemical properties [189,237].

### 4.4. Integrated Mechanistic Model of Fungal Pigment-Mediated Nanoparticle Synthesis

The synthesis of nanoparticles using fungal pigments can be viewed as a comprehensive multistage process influenced by the interactions of pigment redox reactions, metal coordination, and surface stabilization. In this cohesive framework, fungal pigments serve as reducing agents, nucleation controllers, and capping ligands, facilitating the formation of nanoparticles under gentle and environmentally friendly conditions.

First, metal ions engage with electron-rich functional groups found in fungal pigments through coordination and electrostatic forces, forming localized pigment–metal complexes. These complexes act as confined reaction sites that promote effective electron transfer from redox-active pigment components, resulting in the reduction in metal ions and the production of zero-valent metal atoms in the catalytic cycle. When a sufficient concentration of these reduced atoms is achieved, homogeneous nucleation occurs, leading to the formation of stable nanoparticle seeds.

Following the initial formation of nuclei, nanoparticles grow through the precise addition of atoms, and pigment molecules and other secreted biomolecules attach to the developing nanoparticle surfaces. This surface passivation controls the rate of growth, prevents clumping, and determines the ultimate size, shape, and stability of nanoparticles in a colloidal state. Factors such as pH, temperature, and pigment concentration influence each phase of this process by affecting pigment ionization, the redox potential, and the interactions between ligands and metals.

This comprehensive mechanistic framework emphasizes the role of fungal pigments as versatile molecular agents that integrate redox activity and surface modification. This study presents a biologically controlled yet chemically influenced method for the eco-friendly synthesis of nanoparticles. This insight offers a logical foundation for customizing nanoparticle characteristics by precisely adjusting the pigment chemistry and reaction parameters.

## 5. Emerging Applications and Technological Prospects

Nanoparticles synthesized using fungal pigments exhibit multifunctionality owing to their natural origin and surface modification by pigment molecules. Pigment-derived capping layers can enhance physicochemical and biological characteristics, such as colloidal stability, surface charge regulation, and bioactivity. However, the majority of reported applications remain at the laboratory level, suggesting potential technological advancements rather than established industrial methodologies. These functional features enable a wide range of applications in the biomedical, environmental, agricultural, and industrial fields. Table 4 offers a detailed summary of different pigment types and their roles in the synthesis of nanoparticles and their associated applications. While these applications demonstrate potential, their extensive implementation within the industry is impeded by challenges pertaining to scalability, consistency, and cost-effectiveness.

## 6. Toxicity and Environmental Safety Considerations

Although the eco-friendly production of nanoparticles using fungal pigments is praised for minimizing environmental impact and reducing the reliance on harmful chemicals, the natural origin of the reducing and capping agents does not guarantee their safety [265]. It is crucial to thoroughly assess their toxicity and potential environmental hazards before widespread adoption [266].

Nanoparticles, irrespective of their method of synthesis, can possess strong antimicrobial effects but may also pose toxicity risks to nontarget organisms [267]. For example, research indicates that nanoparticles can harm aquatic organisms, such as cyanobacteria, by producing reactive oxygen species, leading to membrane damage and pigment breakdown, which can upset the ecological balance [266]. These impacts underscore the potential environmental hazards associated with the release of nanoparticles.

Evaluations of biological interactions and cytotoxicity, particularly in the context of in vitro and biomedical studies, reveal that inherent particle features, such as size, shape, surface chemistry, and aggregation state, significantly impact their biological interactions and toxicity profiles [268]. For instance, gold nanoparticles coated with biological molecules may exhibit different levels of cytotoxicity based on these characteristics and the assessment methods employed. The absence of standardized, universally recognized protocols complicates the safety evaluation and regulatory approval processes [269]. Additionally, the intricate interactions between nanomaterials and biological molecules, such as biocorona formation within biological systems, influence toxicity outcomes and must be considered when designing safer nanomaterials [270].

It is essential to acknowledge that certain fungal pigments, particularly those associated with mycotoxins, may exhibit inherent toxicity [15]. Various mycotoxins, such as oxaline, secalonic acid D, tanzawaic acid A, aspergiolide A, erythroskyrin, cyclochlorotine, islanditoxin, citrinin, luteoskyrin, and rugulosin are concurrently synthesized in the medium by *Aspergillus* and *Penicillium* species [15]. In Japan and Southeast Asia, Monascus pigments are commercially available and legally sanctioned; however, they are prohibited in the European Union (EU) and the United States (US) due to the potential contamination with citrinin, a metabolite known for its nephrotoxic and hepatotoxic properties [271,272]. Over the past two decades, research on Monascus pigments has predominantly focused on strategies to mitigate citrinin production or on developing strains that do not produce citrinin [273]. Therefore, the safety of nanoparticle systems incorporating pigments is contingent not only upon the intrinsic properties of the nanoparticles but also on the type and purity of the pigments from which they are derived.

Assessing environmental risks is essential because nanoparticles can accumulate and pose long-term ecological toxicity [266]. The varied physicochemical characteristics that confer distinct functionalities to nanoparticles also lead to their persistence in the environment and potentially harmful biological interactions [274]. Reviews highlight the necessity of comprehensive legislation, life-cycle assessments, and a better understanding of environmental fate to prevent unintended consequences [275].

In conclusion, although fungal pigments are natural and biodegradable, their safety cannot be assumed without rigorous evaluation, and their ability to reduce and stabilize nanoparticles necessitates comprehensive testing for cytotoxicity and environmental impact. While sustainable green synthesis methods strive to minimize the use of harmful substances, they must also incorporate detailed toxicological assessments to ensure safety [276,277].

## 7. Challenges and Knowledge Gaps

Despite the growing interest in using fungal pigments for nanoparticle synthesis, several key challenges and knowledge gaps hinder their broader technological adoption. One of the primary concerns is the inherent variability in pigment yield and composition, which arises from genetic differences among fungal strains, the conditions under which they are grown, and the extraction techniques used. This variability directly affects the size, stability, and functional performance of nanoparticles, resulting in inconsistent outcomes across production batches [225].

The absence of standardized protocols for the synthesis, characterization, and toxicity assessment complicates the comparison of studies and the achievement of reproducibility. Variations in reaction conditions, analytical techniques, and biological testing impede the development of reliable structure–function relationships and the ability to benchmark the performance of nanoparticles created through chemical synthesis [278].

Our understanding of the mechanistic interactions between pigments and metals is limited. While it is commonly believed that fungal pigments function as reducing and capping agents, there is a lack of direct experimental evidence to support these claims. Most research relies on indirect indicators, such as shifts in FTIR or changes in UV–Vis spectra, highlighting the necessity for more advanced spectroscopic and molecular-level studies to verify the binding modes, electron transfer routes, and stabilization processes [225,260].

Ultimately, scaling up and implementing these processes at an industrial level presents considerable obstacles. Most of the documented syntheses are limited to small-scale laboratory batch systems, offering little understanding of how to scale the process, its cost efficiency, long-term durability, and waste management strategies. Challenges such as the efficiency of pigment recovery, process control, and adherence to regulations are largely unaddressed, underscoring the necessity for pilot-scale experiments and techno-economic evaluations [7]. These constraints are particularly significant when considering the increasing scale of global fungal biomass production and the imperative for industrial feasibility.

## 8. Future Perspectives and Research Directions

The annual global production of mushrooms has exceeded 35 million tons, underscoring the growing significance of fungi in both food systems and industrial biotechnology. This considerable biomass presents a substantial opportunity to exploit fungal metabolites, such as pigments, for innovative applications, including nanoparticle synthesis [279]. Concurrently, the global biotechnology sector is witnessing an increasing demand for sustainable and bio-based nanomaterials, driven by environmental and regulatory considerations. The expanding influence of nanotechnologies derived from fungal pigments is becoming increasingly apparent within the broader bioeconomy [279].

The synthesis of nanoparticles using fungal pigments exemplifies an intriguing intersection of green chemistry, microbial biotechnology, and nanoscience. Although there has been swift progress, the field is still in its nascent stages, and its growth hinges on overcoming significant biological, chemical, and translational hurdles. Future endeavors should prioritize boosting pigment yield and ensuring compositional uniformity through strain selection, metabolic and genetic engineering, and optimized fermentation techniques [280]. To guarantee reproducibility, scalability, and consistency across batches in pigment-driven nanoparticle synthesis, controlled downstream processing and pigment standardization are crucial [27,281,282].

One of the key research priorities is to develop standardized protocols for synthesis and characterization that combine pigment chemistry with the physicochemical profiling of nanoparticles. This standardization will facilitate meaningful comparisons across different studies and aid in gaining regulatory approval. Concurrently, a more profound understanding of the interactions between pigments and metals, nucleation pathways, and mechanisms of surface stabilization is necessary. To unravel these complex bio-nano interfaces, advanced analytical and molecular techniques, such as in situ spectroscopic methods, omics-driven pathway analysis, and computational modeling, will be crucial [283,284].

From a practical and translational standpoint, it is crucial to systematically assess the long-term toxicity, environmental behavior, and life-cycle effects of pigment-mediated nanoparticles under conditions that mimic real-world exposure [285]. Integrating pilot-scale production, techno-economic evaluations, and sustainability metrics will help close the gap between laboratory synthesis and industrial application [286,287]. Although these advancements are promising, the widespread industrial application of nanoparticle production via fungal pigments is still constrained by challenges related to scalability, process standardization, and economic viability. Together, these research avenues will speed up the creation of safe, scalable, and functionally adaptable fungal pigment-based nanomaterials for use in biomedical, environmental, and industrial fields [288].

## 9. Conclusions

Fungal pigment-driven nanoparticle synthesis is a flexible and eco-friendly method that combines the natural chemistry of pigments with sustainable nanotechnology. Fungal pigments serve a dual role as both reducing and stabilizing agents, allowing the creation of nanoparticles with specific physicochemical characteristics and improved functional capabilities. As discussed in this review, these nanomaterials have wide-ranging applications in the biomedical, environmental, and industrial sectors, including antimicrobial properties, anticancer capabilities, dye degradation, sensing, and surface modification. Despite these benefits, the field is still in its early stages and faces significant challenges, such as pigment variability, understanding of mechanisms, safety assessments, and scalability. Overcoming these obstacles through standardized methods, advanced mechanistic studies, thorough toxicity evaluations, and pilot-scale experiments is crucial for technological advancement. In summary, fungal pigment-based nanomaterials represent a promising category of bio-enabled systems, and ongoing interdisciplinary research will be vital to fully harness their potential in sustainable, application-focused nanotechnology.

## Figures and Tables

**Table 1:** Examples of different pigment-producing fungi, pigments, and classes of compounds.

SN	Fungal Species	Pigment	Class of Compound	Pigment Color	Ref.
1	*Alternaria solani*, *Alternaria porri*, *Alternaria tomatophila*	Altersolanol A	Hydroxyanthraquinone	Yellow	[51,52]
2	*Alternaria* sp. ZJ9-6B	Alterporriol K, Alterporriol L, Alterporriol M, Macrosporin, Dactylario, Tetrahydroaltersolanol B	Anthraquinone, Hydroxyanthraquinone	Red	[53]
3	*Amygdalaria panaeola*	Panaefluorolines A, Panaefluorolines B, Panaefluorolines C	Isoquinoline	Yellowish green	[54]
4	*Aspergillus variecolor*	Variecolorquinone A	Quinone	Yellow	[55]
5	*Aspergillus awamori*	Asperyellone	--------	Yellow, brown	[56,57]
6	*Aspergillus niger*	Aspergillin	--------	Black	[58]
7	*Aspergillus flavus*	Unknown	--------	Red	[15]
8	*Aspergillus sclerotiorum*	Neoaspergillic acid	--------	Yellow	[15]
9	*Aspergillus versicolor*	Asperversin	--------	Yellow	[59]
10	*Aspergillus sulphureus*	Viopurpurin, Rubrosulfin	Naphtoquinones	Purple, Red	[15,60]
11	*Aspergillus fumigatus*	Melanin	1,8- dihydroxynaphthalene	Dark brown	[61]
12	*Aspergillus nidulans*	Emodin	Hydroxyanthraquinone	Yellow	[62,63]
13	*Aspergillus cristatus*	Erythroglaucin	Hydroxyanthraquinone	Red	[64]
14	*Aspergillus glaucus*	Physcion, Aspergiolide B, Erythroglaucin	Hydroxyanthraquinone	Yellow, Red	[62,64,65]
15	*Aspergillus repens*	Erythroglaucin	Hydroxyanthraquinone	Red	[64]
16	*Aspergillus ochraceus*	Xanthomegnin, Viomellein	Dihydroisocoumarin, Quinone	Reddish brown	[66]
17	*Penicillium aculeatum*	Ankaflavine	Azaphilone	Yellow	[67]
18	*Penicillium purpurogenum*	Nglutarylmonascorubramine, Nglutarylrubropunctamine, Purpurogenone, Mitorubrin, Mitorubrinol	Azaphilone	Purple Red	[49,68,69]
19	*Penicillium marneffei*	Monascorubrin	Azaphilone	Orange	[70]
20	*Penicillium bilaii*	Citromycetin, Citromycin, (–)-2,3- Dihydrocitromycetin, (–)-2,3- Dihydrocitromycin	Chromene	Yellow	[71]
21	*Penicillium oxalicum*	Arpink redTM	Anthraquinone	Red	[72,73]
22	*Penicillium sclerotiorum*	Sclerotiorin	--------	Yellow to orange	[74]
23	*Penicillium rubrum*	Mitorubrin	Azaphilone	Yellow	[75]
24	*Phallus multicolor*	Pencolide	Maleimide	Yellow to Orange	[76,77]
25	*Penicillium phoeniceum*	Phoenicin	Toluquinone	Yellow	[78]
26	*Penicillium frequentans*	Questin	Hydroxyanthraquinone	Yellow to Orange-brown	[79]
27	*Fusarium oxysporum*	13-hydroxynorjavanicin, 1,4-naphthalenedione3,8-dihydroxy-5,7- dimethoxy-2-(2- oxopropyl), 5-O-methyljavanicin, 9-Omethylanhydrofusarubin, 5-O-methylsolaniol, 8-O-methylbostrycoidin, 8-Omethylanhydrofusarubinlactol	Naphthoquinone, Anthraquinone	Red, Purple	[15,79]
28	*Fusarium fujikuroi*	Norbikaverin, Bikaverin, β-carotene, 8-O-methylfusarubin	Benzoxanthentrione, Carotenoids, Naphthoquinone	Red	[79,80]
29	*Fusarium culmorum*	Bostrycoidin	Naphthoquinone	Red	[81]
30	*Fusarium sporotrichioides*	β-carotene/Lycopene	--------	Yellow to orange-red	[82]
31	*Fusarium verticillioides*	Napthoquinone, Benzoquinone	-------	Yellow	[15,83]
32	*Monascus purpureus*	Monascorubramine, Monascorubrin, Monapilol A, Monapilol B, Monapilol C, Monapilol D, Monascin, Ankaflavin, Rubropuntatin, Monascopyridine B	Azaphilone	Red, Orange, Yellow	[82,83,84,85,86,87,88,89,90]
33	*Monascus purpureus* FTC 5357	Monascorubramine	--------	Red	[91]
34	*Monascus rubropunctatus*	Rubropunctamine, Rubropunctatin	Azaphilone	Red, Orange	[90]
36	*Monascus ruber* CCT 3802	Monascorubrin	--------	Orange, yellow and red	[92]
37	*Talaromyces funiculosus*	Ravenelin	Xanthone	Yellow	[93]
38	*Talaromyces australis*	--------	--------	Red	[23,24]
39	*Talaromyces atroroseus*	Azaphilone	--------	Red	[82]
40	*Trichoderma harzianum*	Pachybasin, Emodin	Hydroxyanthraquinone	Yellow	[94]
41	*Trichoderma aureoviride*	Chrysophanol	Hydroxyanthraquinone	Orange-red	[79]
42	*Penicillium maximae*	--------	Azaphilones	yellow-orange-red	[95]
43	*Alternaria burnsii* NFCCI 5753	Melanin	--------	Black	[96]
44	*Cladosporium tenuissimum* NFCCI 5754	Melanin	--------	Black	[96]

The notation ‘--------’ indicates that cited literature lacked detailed chemical analysis or clear structural classification of the pigment. This does not suggest these compounds do not exist, but highlights knowledge gaps requiring further phytochemical and spectroscopic research.

**Table 2:** Major fungal carotenoids and their structural features, biological properties, and functional relevance.

Pigment	Chemical Class	Key Structural Features	Representative Fungal Producers	Major Biological/Functional Properties	Ref.
β-Carotene	Carotene (C_40_ terpenoid)	11 conjugated double bonds; oxygen-free	*Blakeslea trispora*, *Mucor circinelloides*, *Phycomyces* spp.	Provitamin A; strong antioxidant; photoprotective	[118,119,120,121,122,123,124,125]
Lycopene	Acyclic carotene	Open-chain polyene; highest number of conjugated double bonds	*Fusarium sporotrichioides*, *Talaromyces amestolkiae*	Potent antioxidant; highly redox-active	[17,126,127,128,129,130]
Canthaxanthin	Xanthophyll	Keto-functional groups; moderate polarity	*Blakeslea trispora*, *Neurospora* spp.	Antioxidant; lipid oxidation inhibitor	[131,132,133]
Astaxanthin	Xanthophyll	Hydroxyl and keto groups at terminal rings	*Xanthophyllomyces dendrorhous*	Exceptional antioxidant; anti-inflammatory; photoprotective	[134,135,136,137,138,139]
Torulene	Carotene	Extended conjugated system; dehydrogenated structure	*Rhodotorula*, *Sporobolomyces*, *Neurospora* spp.	Antioxidant; antimicrobial	[140,141,142]
Torularhodin	Xanthophyll (carboxylated)	Carboxyl group; high polarity	*Rhodotorula glutinis*, *Sporidiobolus* spp.	Strong lipid peroxidation inhibitor	[141,143,144,145]

**Table 3:** Major classes of fungal polyketide pigments, representative producers, and their functional significance.

Pigment	Key Structural Features	Representative Fungal Producers	Key Properties	Ref.
Melanins	Heterogeneous indolic or phenolic polymers; high molecular weight	*Aspergillus*, *Cryptococcus*, *Magnaporthe*, *Colletotrichum* spp., *Alternaria burnsii* NFCCI 5753, *Cladosporium tenuissimum* NFCCI 5754	Broad-spectrum antioxidant, UV-shielding, antimicrobial, redox-active	[28,96,152,153,154]
Anthraquinones	Tricyclic quinonoid structures derived from octaketides	*Aspergillus*, *Penicillium*, *Fusarium*, *Eurotium* spp.	Color diversity (yellow–red), redox-active, bio-safe colorants	[72,109,155]
Hydroxyanthraquinones	Hydroxyl-substituted anthraquinones (e.g., emodin, physcion)	*Penicillium oxalicum*, *Aspergillus glaucus*	High antioxidant activity, commercial food colorants	[64,156,157]
Azaphilones	Oxygenated pyranoquinone bicyclic core	*Monascus*, *Talaromyces*, *Chaetomium*, *Penicillium* spp., *Penicillium maximae*	pH- and heat-stable pigments; strong bioactivity	[95,158,159,160,161]
Quinones	Conjugated cyclic diketones; age-dependent pigmentation	*Aspergillus*, *Penicillium*, *Helminthosporium* spp.	Strong redox cycling; chromatic variability	[162,163,164]
Naphthoquinones	Fused aromatic quinones structurally related to shikonin	*Cordyceps unilateralis*, *Epicoccum nigrum*	Light-, heat-, and pH-stable red pigments	[165,166]

**Table 4:** Applications of fungal pigment–mediated nanoparticles synthesized via green routes.

SN	Fungal sp.	Pigment	Reaction Condition	Types of NPs	Size (nm)	Shape	Applications	Ref.
1	*M. purpureus* NRRL 1992	Monascus pigment (mixture)	The cell filtrate was challenged with AgNO_3_ (1 mM) and agitated at 25 °C in a dark place	AgNPs	1–7	Spherical to cuboidal	Antibacterial, Antifungal	[255]
2	*M. purpureus* NMCC-PF01	Monascus orange and red pigment	100 µL (mg/mL) of pigment mixed with 2.5 mL AuCl_3_ under sunlight	AuNPs	10–60	Spherical, triangular	NT	[256]
3	*M. purpureus* NMCC-PF01	Red Monascus pigment	100 µL (mg/mL) mixed with 2.5 mL AgNO_3_ 0.2 mM under sunlight	AgNPs	10–40	Spherical	Antibacterial, Antibiofilm, and colorimetric metal sensing	[224]
4	*M. ruber* C100	Monascus pigment (mixture)	2 mL of pigment mixed with 20 mL of 0.2 mM AgNO_3_ under xenon light radiation 500 W	AgNPs	18	Spherical	Antioxidant, Antibacterial, Dye degradation	[257]
5	*Talaromyces purpurogenus*	A mixture of red, orange, and yellow pigments	0.5 g/L pigment mixed with 5 mL of 2 mM AgNO_3_ (pH12) at 28 °C with 2000lx of light for 48 h	AgNPs	4–41	Spherical, hexagonal, rod-shaped	Antibacterial and Anticancer	[258]
6	*Thermomyces* sp.	Yellow pigment	100 mL of filtrate mixed with 1 mM of AgNO_3_ cultured for a day in dark	AgNPs	10–50	Spherical	Textile applications	[259]
7	*Talaromyces purpurogenum*	Mixture of pigment red, orange, and yellow	0.5 g/L pigment incubated with 2 mM of AgNO_3_ at pH 10 exposed in diff. lights	AgNPs	4–41	Spherical, hexagonal, rod-shaped	Antibacterial and anticancer	[260]
8	*Y. lipolytica* NCYC789	Melanin	Chloroauric acid diff. concentrations incubated with 1 mL of melanin	AuNPs	Not specify	Hexagonal, triangular	Antibacterial	[261]
9	*Y. lipolytica* NCIM3589	Melanin	150 mg melanin and 2.5 mM of gold salt in 10 mL incubated at 100 °C for 10 min	AuNPs	Not specify	Not specify	Antimicrobial, antibiofilm	[262]
10	*Penicillium chrysogenum*	Melanin	9.535 mg/mL melanin mixed with 4 mM MgNO_3_ in 1:2 ratio isopropanol as radical controller exposed to gamma rays	MgO-NPs	5–12	Spherical	Antimicrobial	[263]
11	*Monascus* sp. FZU04	Rubropunctain	3 mL of pigment mixed with 15 mL 0.001 mM AgNO_3_ at 80 °C oil bath for 60 min	AgNPs	13.54	Round	Antibacterial	[264]

## Data Availability

No new data were created or analyzed in this study. Data sharing is not applicable to this article.
